# Supplementary material for: Responses of Three Pedicularis Species to Geological and Climatic Changes in the Qinling Mountains and Adjacent Areas in East Asia
Source: Plants (Basel). 2024 Mar 8;13(6):765. doi: 10.3390/plants13060765 (PMC10974801; doi:10.3390/plants13060765)
Supplement: Supplementary file 1 [file plants-13-00765-s001.zip › plants-2872110-supplementary.pdf]

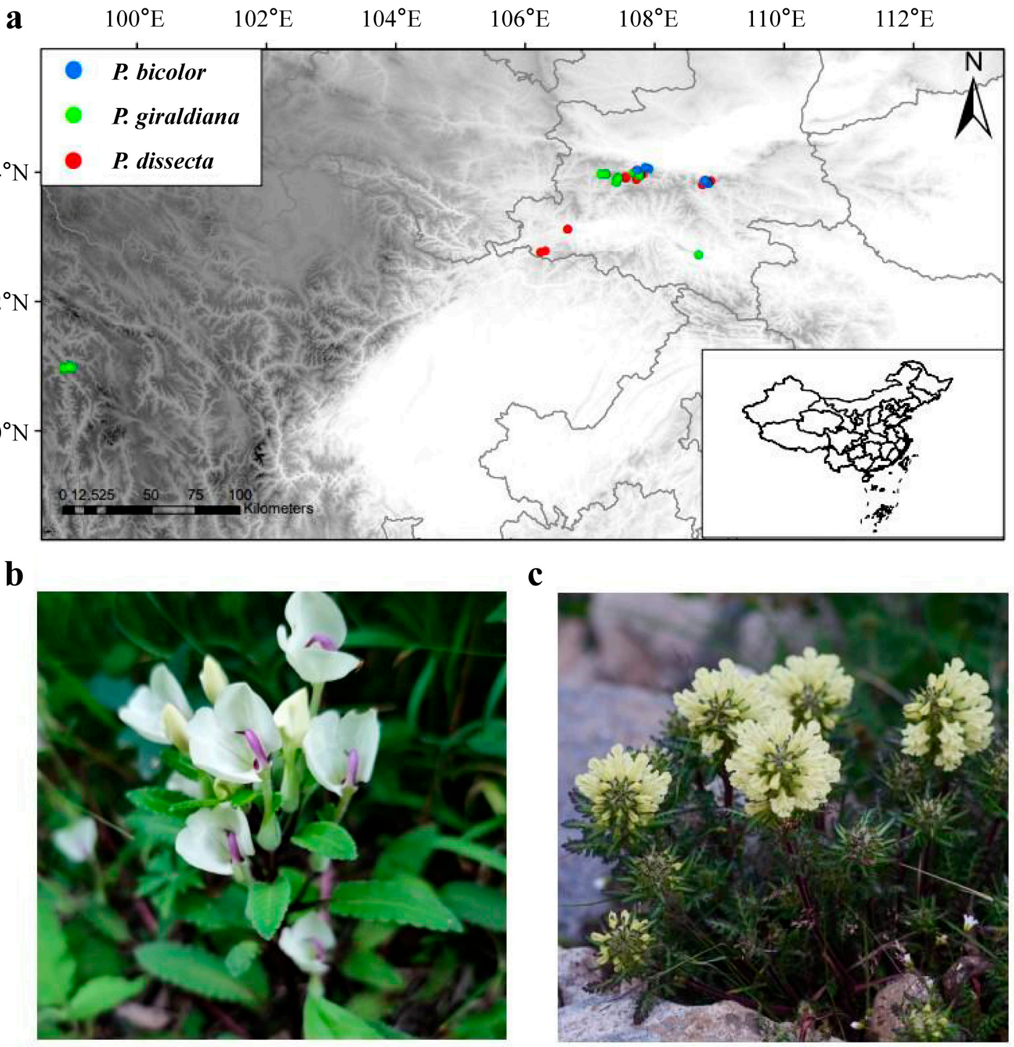

Figure S1. Geographical distribution of sampled populations of three *Pedicularis* species. (a) blue, green, and red indicate *P. bicolor*, *P. dissecta* and *P. giraldiana*, respectively. Photographs of representative individuals of (b) *P. bicolor* and (c) *P. giraldiana*

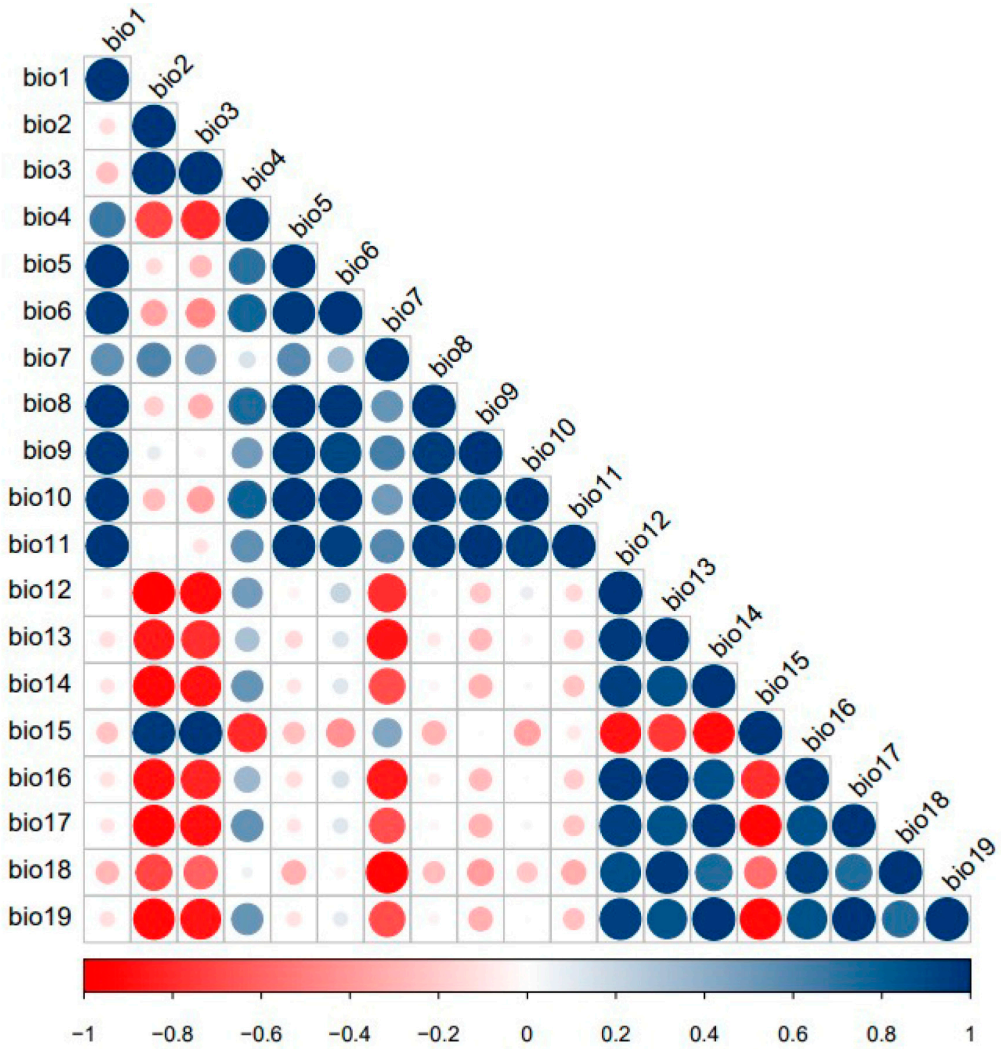

Figure S2. Correlation analysis of various environmental factors. Red represents positive correlations and blue represents negative correlations.

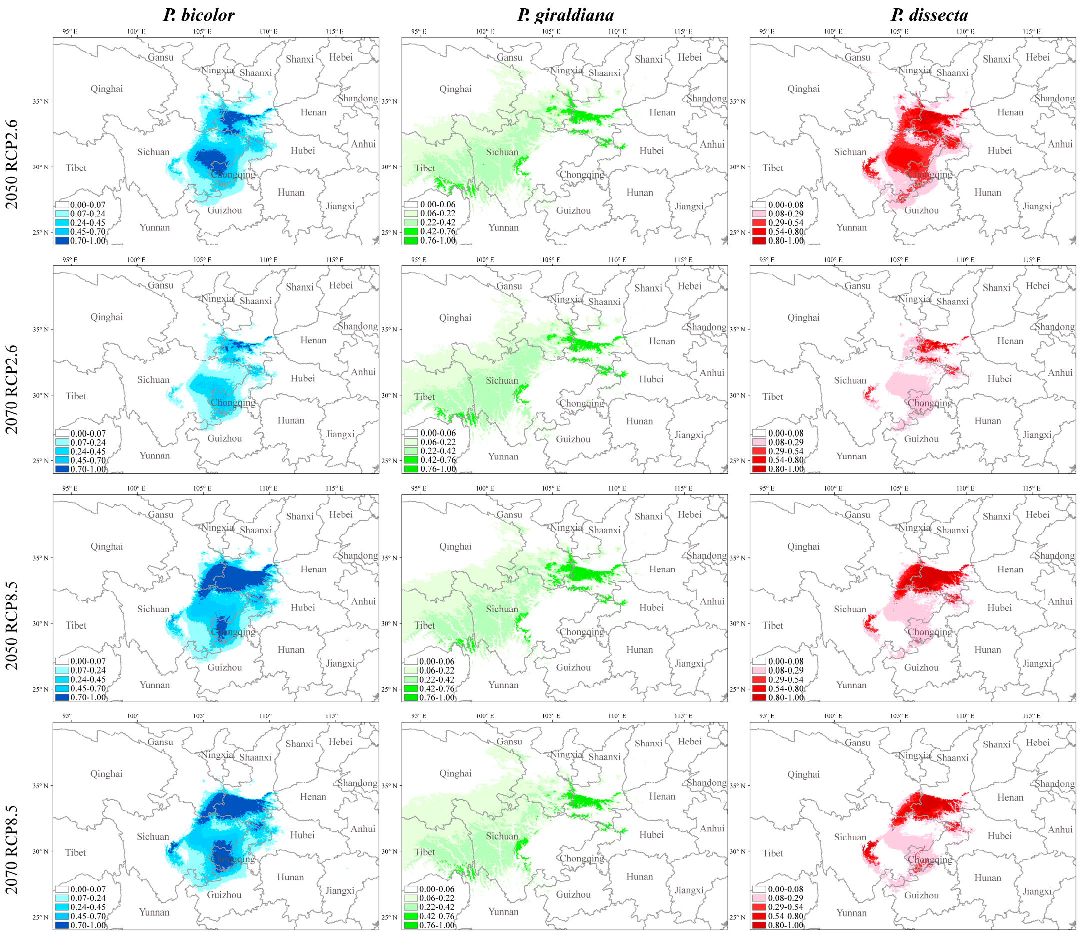

Figure S3. Prediction of potential geographical distributions of three *Pedicularis* species in different climatic scenarios in the future based on ecological niche modeling.

Table S1. Taxa and GenBank accessions used in phylogeny and divergence time analysis.

Taxon	Accession	Chloroplast Genome (bp)
Pedicularis cheilanthifolia	MT040754	147,807
Pedicularis longiflora	NC_046852	153,547
Pedicularis verticillata	MT040753	142,733
Pedicularis hallaisanensis	NC_037433	143,469
Pedicularis muscicola	NC_046853	152,907
Pedicularis resupinata	NC_046397	153,145
Pedicularis oederi	NC_046854	153,139
Pedicularis oederi var. sinensis	MW770457	153,770
Pedicularis dissecta	NC_056312	152,120
Pedicularis shansiensis	NC_058762	151,902
Pedicularis ishidoyana	NC_029700	152,571
Orobanche pancicii	NC_031443	88,525
Orobanche crenata	NC_024845	87,529
Orobanche rapum-genistae	NC_031444	91,517
Orobanche densiflora	NC_031442	83,024
Siphocranion macranthum	MT473779	152,126
Siphocranion flavidum	MT473778	152,039
Scutellaria baicalensis	NC_027262	152,731

Table S2. Environmental variable information of distribution area prediction.

Type	Variables	Descriptions	Units
Climate	bio1	Annual Mean Temperature	◦C
	bio2	Mean Diurnal Range (Mean of monthly (max temp-min temp))	◦C
	bio3	Isothermality (bio2/bio7) (×100)	/
	bio4	Temperature Seasonality (standard deviation ×100)	/
	bio5	Max Temperature of Warmest Month	◦C
	bio6	Min Temperature of Coldest Month	◦C
	bio7	Temperature Annual Range (bio5-bio6)	◦C
	bio8	Mean Temperature of Wettest Quarter	◦C
	bio9	Mean Temperature of Driest Quarter	◦C
	bio10	Mean Temperature of Warmest Quarter	◦C
	bio11	Mean Temperature of Coldest Quarter	◦C
	bio12	Annual Precipitation	mm
	bio13	Precipitation of Wettest Month	mm
	bio14	Precipitation of Driest Month	mm
	bio15	Precipitation Seasonality (Coefficient of Variation)	/
	bio16	Precipitation of Wettest Quarter	mm
	bio17	Precipitation of Driest Quarter	mm
	bio18	Precipitation of Warmest Quarter	mm
	bio19	Precipitation of Coldest Quarter	mm
